# Case report: Methicillin-resistant *Staphylococcus aureus* with penicillin susceptible (PS-MRSA): first clinical report from a psychiatric hospital in China

**DOI:** 10.3389/fmed.2024.1380369

**Published:** 2024-04-04

**Authors:** Fei Yan, Mi Yang, Yuting Sun, Qin Tang, Lu Yuan

**Affiliations:** ^1^Department of Clinical Laboratory, The Fourth People’s Hospital of Chengdu, Chengdu, China; ^2^Department of Clinical Laboratory, The Clinical Hospital of Chengdu Brain Science Institute, MOE Key Lab for Neuroinformation, University of Electronic Science and Technology of China, Chengdu, China

**Keywords:** *Staphylococcus aureus*, MRSA, PS-MRSA, Alzheimer’s disease, penicillin, mecA

## Abstract

This case report documents the first instance of Penicillin-Susceptible Methicillin-Resistant *Staphylococcus aureus* (PS-MRSA) in a Chinese psychiatric hospital. The strain was isolated from a patient with Alzheimer’s disease who had a lower respiratory tract infection. Clinical and laboratory analyses, including mass spectrometry, antibiotic susceptibility testing, and whole-genome sequencing, confirmed the PS-MRSA strain. In this case, we systematically introduce the clinical symptoms, laboratory findings, and treatment responses associated with this PS-MRSA strain. This discovery offers a new perspective on our understanding of resistance mechanisms and expands our considerations for existing antibiotic treatments. It may fill a gap in the classification of MRSA strains, enhance the spectrum of MRSA resistance, and complete the therapeutic strategies for MRSA.

## 1 Introduction

**Figure 1:**
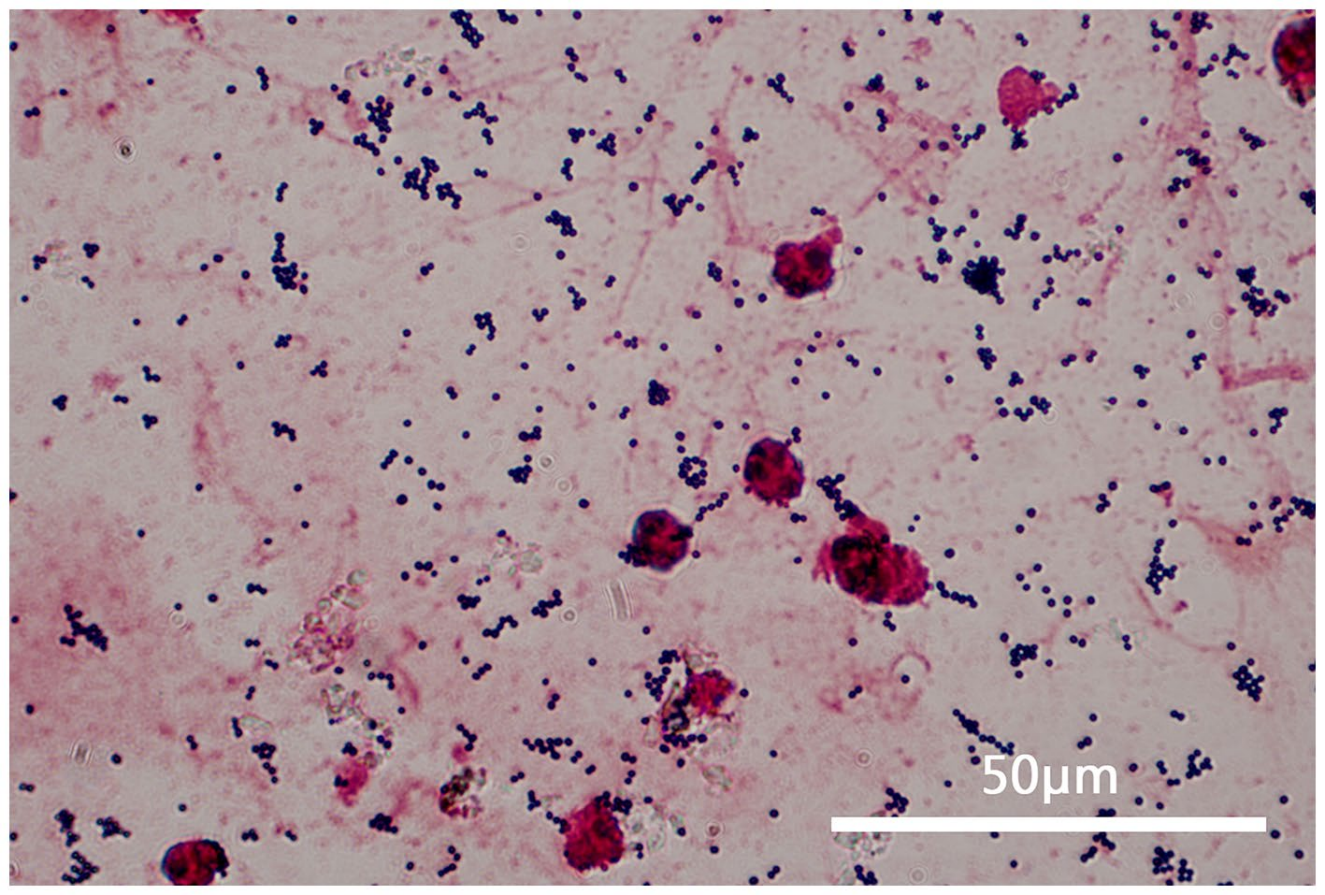
This figure shows a sample of the patient’s sputum at 1000 times magnification under a microscope after gram staining. It can be observed that the sputum specimen is a qualified specimen (white blood cells >25 and epithelial cells <10), and gram-stained positive cocci can be seen all over the visual field, in a grape-like arrangement.

**Figure 2:**
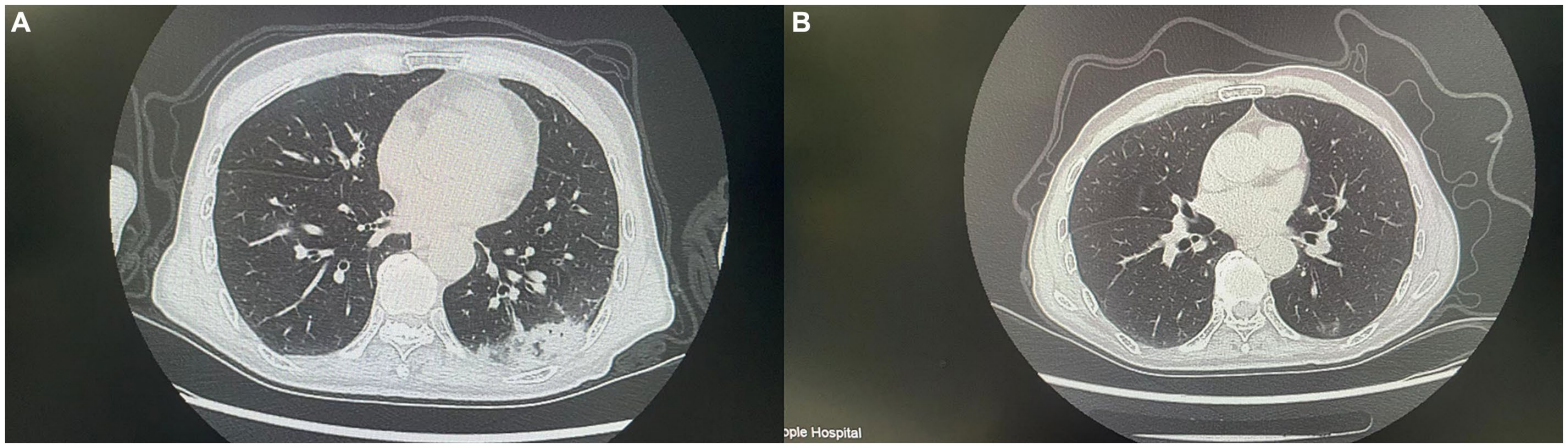
This figure shows CT images of the patient before and after antibiotic treatment. **(A)** (Before treatment) shows increased density in multiple areas of the lungs. **(B)** Shows a significant reduction in the inflammatory area and clearer lung structure.

**Figure 3:**
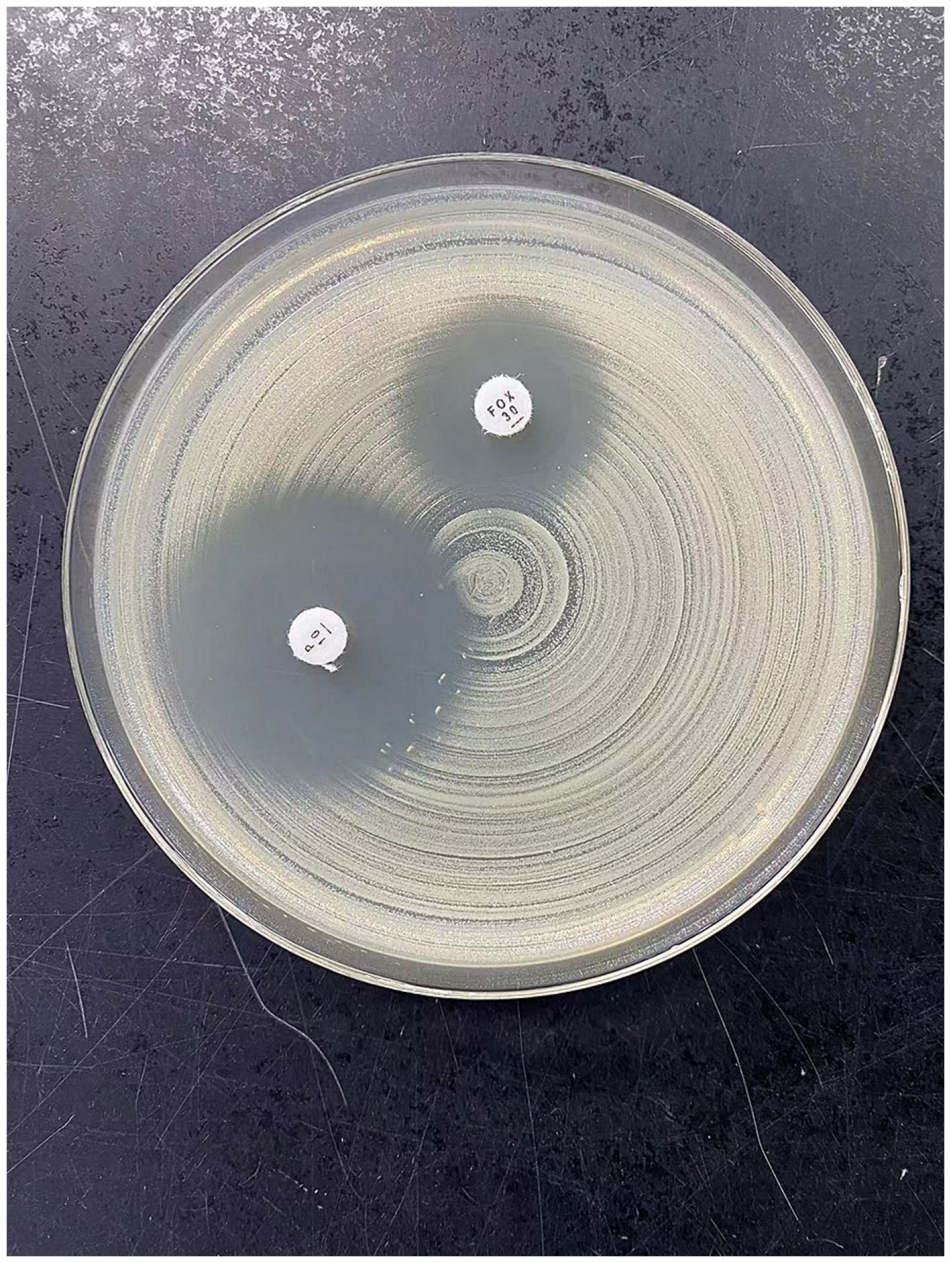
This figure shows the results of PS-MRSA after disk susceptibility test using MH plate. Antibacterial zone diameter of cefoxitin: 15 mm (drug resistance); beach-like changes in the penicillin edge test.

Methicillin-resistant *Staphylococcus aureus* (MRSA) is a bacterium resistant to conventional penicillin antibiotics. Resistance primarily occurs due to the PBP2a protein’s low affinity for β-lactam antibiotics, a protein encoded by the mecA gene (1). While oxacillin-susceptible MRSA (OS-MRSA) is common worldwide (2), penicillin-susceptible MRSA (PS-MRSA) has not previously been reported in China. Literature reports a PS-MRSA strain causing nosocomial infections in a Japanese pediatric hospital (3), underscoring the importance of preventing hospital-acquired infections. This case report focuses on the clinical manifestations, characteristics, and drug treatment process of patients with PS-MRSA, integrating laboratory results such as genome sequencing to comprehensively analyze the occurrence mechanism of this PS-MRSA strain. Given the rarity of PS-MRSA, especially in China, the following section details the clinical presentation of a patient with PS-MRSA, highlighting the unique aspects of this case and its implications for treatment in a psychiatric hospital setting (see Figures 1–3).

## 2 Case description

The patient, a female, was admitted to a tertiary psychiatric specialty hospital for Alzheimer’s disease. In April 2023, she began showing symptoms of a lower respiratory tract infection, such as cough, sputum production, and fever. Laboratory tests showed a white blood cell count of 6.11 × 10^9^/L, a neutrophil percentage of 81.5%, a hypersensitive C-reactive protein (hs-CRP) level of 50.40 mg/L, and a procalcitonin level of 0.155 ng/mL. Blood gas analysis indicated type II respiratory failure. Chest CT scans revealed scattered inflammation in both lungs. Sputum culture identified MRSA, and symptoms improved after vancomycin treatment. By the end of May 2023, the patient’s symptoms worsened, with a white blood cell count of 7.60 × 10^9^/L, a neutrophil percentage of 61.9%, a hs-CRP level of 166.88 ng/L, and a procalcitonin level of 0.255 ng/mL. CT scans showed increased pulmonary inflammation. Sputum culture then identified PS-MRSA, and symptoms improved following treatment with ceftriaxone. In early January 2024, the patient experienced another lower respiratory tract infection, with a white blood cell count of 13.55 × 10^9^/L/L, a neutrophil percentage of 80.8%, a hs-CRP level of 113.93 mg/L, and a procalcitonin level of 0.29 ng/mL. CT scans indicated increased pulmonary inflammation. After empirically treating with penicillin for 3 days, symptoms alleviated, infection markers decreased, and sputum culture again detected PS-MRSA. The clinical progression of the patient’s condition necessitated detailed laboratory testing to accurately identify the strain and its antibiotic susceptibility. The laboratory test included collecting samples from the patient’s sputum after oral cleaning and confirming the presence of Gram-positive cocci via microscopic examination. The strain was identified as *Staphylococcus aureus* using the bioMérieux VITEK 2 and Autof MS1000 systems. Antibiotic susceptibility testing was performed with the bioMérieux VITEK 2 automated system and the disc diffusion method (Wenzhou Kangtai Biological Technology Co., Ltd., Zhejiang medical device registration number 20152400139), confirming the strain’s sensitivity to penicillin and resistance to cefoxitin. Finally, the strain was sent to Shanghai Majorbio Bio-pharm Technology Co., Ltd. for *de novo* genome sequencing to analyze its resistance-related genes.

## 3 Discussion

This case of PS-MRSA challenges the typical avoidance of β-lactam antibiotics for MRSA treatment (4), a strategy usually adopted due to the PBP2a protein production by the mecA gene. Uniquely, the empirical use of β-lactam antibiotics (ceftriaxone and penicillin) in this case resulted in effective therapeutic responses. This may suggest variations in the expression or function of the mecA gene among different MRSA strains, or that PS-MRSA might possess a resistance mechanism different from conventional MRSA (5). This case suggests that the mecA gene’s expression level is not strictly proportional to resistance (6), as PS-MRSA exhibited low-level resistance to oxacillin. This could be due to a possible mutation or downregulation of the mecA gene, affecting the PBP2a protein function (7). Further whole-genome sequencing of PS-MRSA revealed low identity (%) of PBP-related genes, indicating that the consistency between sequencing data and the entire reference genome is low (Supplementary material). This reveals the presence of heterogeneity in resistance genes even within MRSA strains. Such heterogeneity could lead to significant differences in sensitivity to the same class of antibiotics among different strains. This finding suggests that in studying resistance, we should not only focus on the gene itself but also consider the regulation of gene expression and its impact on protein function (8).

According to the rules of the Chinese Ministry of Health on antibiotic use in psychiatric hospitals, some broad-spectrum antibiotics are not allowed to be used in clinical treatment. Previous studies have shown that certain psychotropic drugs can enhance the antibiotic effect against MRSA (9). Future research could explore the application of drug combinations or new therapies in restoring or enhancing antibiotic efficacy. The exploration of such integrated treatment strategies may provide more effective means for clinical therapy. Therefore, treatment strategies need to consider the specific circumstances of the patient, such as mental state, comorbidities, and medications used. When formulating treatment plans, these factors should be comprehensively considered for their impact on drug responses and potential drug interactions (10).

Our laboratory conducted comprehensive antibiotic susceptibility testing on all clinically isolated *Staphylococcus aureus* samples in this case study. In addition to standard testing with automated susceptibility testing instruments, we specifically included disc diffusion tests for penicillin and cefoxitin. This comprehensive approach to antibiotic susceptibility testing enabled the timely identification of this rare PS-MRSA strain, highlighting the importance of employing diverse susceptibility testing methods in resistance research and the critical role of precise diagnosis in antibiotic treatment (11). Although the empirical use of β-lactam antibiotics in this case was effective, it also serves as a reminder of the broader implications of antibiotic use and the development of resistance. Irrational use of antibiotics can accelerate the development of resistance, thus necessitating global cooperation and surveillance to effectively manage antibiotic resistance (12).

Multiple sampling tests conducted on the patient and their surrounding environment did not detect the spread of PS-MRSA, suggesting that it may be confined to the patient herself, with no evidence of widespread transmission in the hospital. This underscores the need for ongoing environmental monitoring and stringent infection control to prevent nosocomial infections (13), particularly in high-risk settings like psychiatric specialty hospitals.

In conclusion, this PS-MRSA case in a Chinese psychiatric hospital highlights the complexities of managing MRSA and its resistance patterns, underscoring the need for specialized research and vigilance. It highlights the need for continued research and vigilance in antibiotic usage and resistance monitoring, particularly in specialized healthcare settings.

## Data availability statement

The datasets presented in this study can be found in online repositories. The names of the repository/repositories and accession number(s) can be found in the article/Supplementary material.

## Ethics statement

The studies involving humans were approved by Ethics Committee of Chengdu Fourth People’s Hospital/partnership. The studies were conducted in accordance with the local legislation and institutional requirements. The participants provided their written informed consent to participate in this study. The manuscript presents research on animals that do not require ethical approval for their study. Written informed consent was obtained from the individual(s) for the publication of any potentially identifiable images or data included in this article.

## Author contributions

FY: Writing – original draft, Writing – review & editing. MY: Writing – original draft, Writing – review & editing. YS: Conceptualization, Data curation, Formal analysis, Funding acquisition, Investigation, Methodology, Project administration, Resources, Software, Supervision, Validation, Visualization, Writing – review & editing. QT: Conceptualization, Data curation, Formal analysis, Funding acquisition, Investigation, Methodology, Project administration, Resources, Software, Supervision, Validation, Visualization, Writing – original draft. LY: Conceptualization, Data curation, Formal analysis, Funding acquisition, Investigation, Methodology, Project administration, Resources, Software, Supervision, Validation, Visualization, Writing – review & editing.

## References

[1] Peacock SJ Paterson GK. Mechanisms of methicillin resistance in *Staphylococcus aureus*. Annu Rev Biochem. (2015) 84:577–601. doi: 10.1146/annurev-biochem-060614-034516, PMID: 26034890

[2] Sabat AJ Pournaras S Akkerboom V Tsakris A Grundmann H Friedrich AW. Whole-genome analysis of an oxacillin-susceptible CC80 mecA-positive *Staphylococcus aureus* clinical isolate: insights into the mechanisms of cryptic methicillin resistance. J Antimicrob Chemother. (2015) 70:2956–64. doi: 10.1093/jac/dkv210, PMID: 26198147

[3] Minami K Terakawa R Sato M Shoji Y Hiroma T Nakamura T. A colonization outbreak of penicillin-susceptible mecA-positive *Staphylococcus aureus* in a neonatal ward of children’s hospital. Infect Control Hosp Epidemiol. (2018) 39:239–41. doi: 10.1017/ice.2017.266, PMID: 29332612

[4] Ersoy SC Abdelhady W Li L Chambers HF Xiong YQ Bayer AS. Bicarbonate resensitization of methicillin-resistant *Staphylococcus aureus* to β-lactam antibiotics. Antimicrob Agents Chemother. (2019) 63:e00496. doi: 10.1128/AAC.00496-19, PMID: 31010857 PMC6591647

[5] Ryffel C Kayser FH Berger-Bächi B. Correlation between regulation of mecA transcription and expression of methicillin resistance in staphylococci. Antimicrob Agents Chemother. (1992) 36:25–31. doi: 10.1128/AAC.36.1.25, PMID: 1375449 PMC189220

[6] Parvez MAK Shibata H Nakano T Niimi S Fujii N Arakaki N. No relationship exists between PBP 2a amounts expressed in different MRSA strains obtained clinically and their β-lactam MIC values. J Med Investig. (2008) 55:246–53. doi: 10.2152/jmi.55.246, PMID: 18797139

[7] Alhadrami HA Hamed AA Hassan HM Belbahri L Rateb ME Sayed AM. Flavonoids as potential anti-MRSA agents through modulation of PBP2a: a computational and experimental study. Antibiotics. (2020) 9:562. doi: 10.3390/antibiotics9090562, PMID: 32878266 PMC7559925

[8] Vestergaard M Frees D Ingmer H. Antibiotic resistance and the MRSA problem. Microbiol Spectr. (2019) 7. doi: 10.1128/microbiolspec.GPP3-0057-2018 PMC11590431 30900543

[9] Wassmann CS Lund LC Thorsing M Lauritzen SP Kolmos HJ Kallipolitis BH. Molecular mechanisms of thioridazine resistance in *Staphylococcus aureus*. PLoS One. (2018) 13:e0201767. doi: 10.1371/journal.pone.0201767, PMID: 30089175 PMC6082566

[10] Gonzales PR Pesesky MW Bouley R Ballard A Biddy BA Suckow MA. Synergistic, collaterally sensitive β-lactam combinations suppress resistance in MRSA. Nat Chem Biol. (2015) 11:855–61. doi: 10.1038/nchembio.1911, PMID: 26368589 PMC4618095

[11] Skov R Lonsway DR Larsen J Larsen AR Samulioniené J Limbago BM. Evaluation of methods for detection of β-lactamase production in MSSA. J Antimicrob Chemother. (2021) 76:1487–94. doi: 10.1093/jac/dkab032, PMID: 33615356 PMC9479532

[12] Garoy EY Gebreab YB Achila OO Tekeste DG Robel RK Ruta GK. Methicillin-resistant *Staphylococcus aureus* (MRSA): prevalence and antimicrobial sensitivity pattern among patients—a multicenter study in Asmara Eritrea. Can J Infect Dis Med Microbiol. (2019) 2019:8321834. doi: 10.1155/2019/8321834, PMID: 30881532 PMC6381584

[13] Turner NA Sharma-Kuinkel BK Maskarinec SA Eichenberger EM Shah PP Carugati M. Methicillin-resistant *Staphylococcus aureus*: an overview of basic and clinical research. Nat Rev Microbiol. (2019) 17:203–18. doi: 10.1038/s41579-018-0147-4, PMID: 30737488 PMC6939889

